# Lateral retinacular release combined with MPFL reconstruction for patellofemoral instability: a systematic review

**DOI:** 10.1007/s00402-020-03689-9

**Published:** 2020-12-14

**Authors:** Filippo Migliorini, Nicola Maffulli, Jörg Eschweiler, Valentin Quack, Markus Tingart, Arne Driessen

**Affiliations:** 1 Department of Orthopaedics, University Clinic Aachen, RWTH Aachen University Clinic, Pauwelsstraße 30, 52074 Aachen, Germany; 2 Department of Medicine, Surgery and Dentistry, University of Salerno, Via S. Allende, 84081 Baronissi, SA Italy; 3 School of Pharmacy and Bioengineering, Keele University School of Medicine, Thornburrow Drive, Stoke on Trent, England; 4 Barts and the London School of Medicine and Dentistry, Centre for Sports and Exercise Medicine, Queen Mary University of London, Mile End Hospital, 275 Bancroft Road, London, E1 4DG England

**Keywords:** Patellofermoral instability, Dislocations, MPFL reconstruction, Lateral retinacular release

## Abstract

**Introduction:**

The role of the lateral retinaculum in patellofemoral instability is still debated. Lateral retinacular release (LRR), has been extensively performed in combination with different surgical procedures, including reconstruction of medio-patellofemoral ligament (MPFL). Despite controversial indications, the results from these studies seem promising. The present study conducts a systematic review about current biomechanical and clinical evidence concerning the role of LRR in combination with MPFL reconstruction. We performed a comprehensive literature research, comparing the outcomes of MPFL reconstruction with and without LRR.

**Materials and methods:**

This systematic review was conducted according to the PRISMA guidelines. The literature search was performed in August 2020. All articles describing the outcome of isolated MPFL reconstruction alone or in combination with a LRR in patients with recurrent patellofemoral instability were considered for inclusion. Only articles reporting data on patients with a minimum of 12-month follow-up were included. Only articles reporting quantitative data under the outcomes of interest were included.

**Results:**

A total of 63 articles were eligible for this systematic review, including 2131 knees. The mean follow-up was 40.87 ± 24.1 months. All scores of interests improved in favour of the combined group: Kujala + 3.8% (*P* = 0.01), Lysholm + 4.2% (*P* = 0.004), Tegner + 0.8 points (*P* = 0.04), IKDC + 9.8% (*P* = 0.02). The ROM was comparable between the two groups (*P* = 0.4). Similarity was found in terms of positivity to the apprehension test (*P* = 0.05), rate of complications (*P* = 0.1), re-dislocations (*P* = 0.8), and revision surgeries (*P* = 0.1).

**Conclusion:**

There is no evidence that adding a lateral release impacts positively on the outcome of MPFL reconstruction.

*Level of evidence*: IV, Systematic review

## Introduction

Patellofemoral instability is a complex disorder with higher prevalence in young and active individuals [1, 2]. The aetiology of patellofemoral instability is multifactorial [3, 4]. Several risk factors have been described that synergistically contribute to joint instability [5, 6]. Most patients present a combination of two or more risk factors [7, 8]. Irrespective of the complexity of etiological causes, with the lateral dislocation of the patella, the syndrome becomes clinically apparent [8]. Several soft tissue and bony stabilizers are involved to ensure a physiological patellar tracking [9]. The bony anatomy of the patella and the trochlea is the most important static stabilizers [10]. Further dynamic restrains are the quadriceps and patellar tendon, the lateral retinaculum, and the medial patellofemoral ligament (MPFL) [9]. The MPFL is the most vital dynamic restrain to patellar lateralization during the first 30° of flexion [11]. After the first event of patellar dislocation, the MPFL is practically always damaged [12]. MPFL reconstruction yields a high success in reducing the rate of further re-dislocations, restoring patients’ quality of life and participation in recreational activities [13]. Conversely, the role of lateral retinaculum in patellofemoral instability remains unclear and debated [14, 15]. Furthermore, lateral retinacular release (LRR) has been extensively performed in combination with various surgical interventions, including MPFL reconstruction [16–33]. Results from studies analysing results after MPFL reconstruction seem promising, but are also criticised for heterogeneity in indication and uncertain evidence [34, 35].

The present systematic review analyses current biomechanical and clinical evidence regarding the role of LRR in combination with MPFL reconstruction. The focus of the present study was on patient-reported outcome measures (PROMs), clinical examination, and complications.

## Materials and methods

### Search strategy

This systematic review was conducted in accordance with the Preferred Reporting Items for Systematic Reviews and Meta-Analyses (PRISMA) [36]. The PICO algorithm guided the initial search:

- P (population): patellofemoral instability;
- I (intervention): isolated MPFL reconstruction;
- C (comparison): combined MPFL reconstruction with LRR;
- O (outcomes): PROMs, clinical examination, complications.

### Literature search

Two authors (JE, FM) independently performed the literature search accessing the following databases Pubmed, Google Scholar, Embase, Scopus in August 2020. The following keywords were used for the search: *patellofemoral instability, recurrent, dislocations, luxation combined with MPFL reconstruction, lateral retinacula, lateral release, re-dislocation, Kujala, Lyshom*. If title matched the topic, the abstract was read and the full text of the article was accessed. The bibliographies of the articles of interest were screened by hand. Disagreements between the author were debated and mutually solved.

### Eligibility criteria

All articles about treatment of patellofemoral instability with an isolated MPFL reconstruction or in combination with lateral retinacula release were considered for inclusion. Articles’ level of evidence I to IV, according to the Oxford Centre of Evidenced-Based Medicine [37], was included in the present work. Given the authors’ language capabilities, articles in English, Italian, French, Spanish, and German were considered for inclusion. Only studies investigating patients affected by recurrent dislocations were eligible. Articles reporting treatment in patients with acute, congenital or habitual dislocations were excluded. Articles about the treatment of patellofemoral instability after total knee arthroplasty were excluded. Techniques, comments, letters, editorials, protocols, and guidelines were excluded. Biomechanical, animal, and cadaveric studies were also excluded. Articles reporting a follow-up < 12 months were excluded. Articles reporting patellofemoral outcomes in revision setting were excluded. Articles combining MPFL reconstruction with bony procedures (trochleoplasty, Elmslie Trillat, Maquet, Fulkerson, Roux–Goldthwait, rotational osteotomies) were excluded. Articles combining MPFL reconstruction with other soft tissue procedures rather than the LRR (medial retinacular plication, ligament plasties, MPFL suture, muscle advancements, or tendon transfer) were excluded. Only articles reporting quantitative data under the outcomes of interest were included. Disagreements between the authors were debated and solved by a third author (MT).

### Outcomes of interest

The following data were collected: generalities (author, year, type of study), demographic baseline (number of samples, mean age), mean follow-up, presence of previous intervention, presence of patho-anatomical risk factors (patella alta, elevated TT-TG, trochlear dysplasia), type of graft, and fixation. The following outcomes of interest were collected: Kujala Anterior Knee Pain Scale [28], Lysholm Knee Scoring Scale [29], Tegner Activity Scale [38], IKDC [39], range of motion (ROM). The same authors also collected clinical examinations, complications, revisions and re-dislocations.

### Methodological quality assessment

For the methodological quality assessment, we referred to the PEDro score. Two authors (FM, JE) independently scored each included article. This score analyzed each article included using the following criteria: clear eligibility criteria, allocation, randomization and blinding methods, follow-up duration, analysis of variables, and intention to treat. The final scoring resulted in a value ranking from 0 (poor quality) to 10 (excellent quality).

### Statistical analysis

Statistical analysis was performed using the IBM SPSS Software version 25. For continuous variables, the mean difference (MD) effect measure standard deviation and T-test were evaluated. For binary data, the odd ratio (OR) effect measure was performed, with confidence interval (CI) set at 95% and chi-quare test. Values of P < 0.05 were considered statistically significant.

## Results

### Search result

**Fig. 1:**
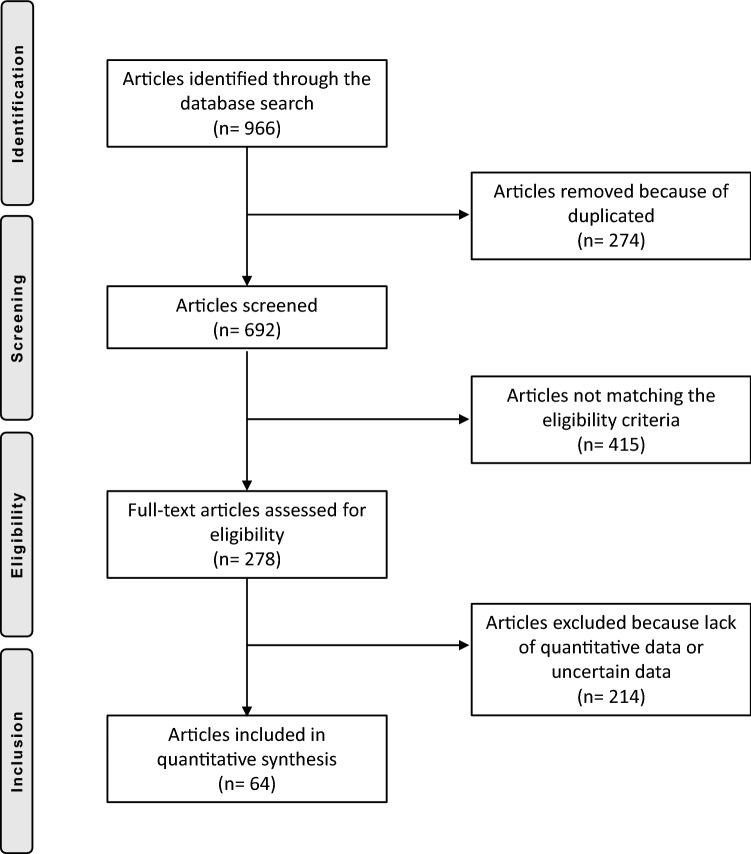
PRISMA flow chart of the literature search

The initial literature search resulted in 966 articles, of which 274 were excluded because of redundancy. Another 415 articles were excluded because they did not match the eligibility criteria; a further 203 articles did not report quantitative data under the endpoints of interest. An additional 11 publications were rejected because they reported uncertain data. Thus, a total of 63 articles were eligible for this systematic review: 24 reporting about MPFL reconstruction in combination with LRR and 40 reporting about isolated MPFL reconstruction. The flow chart of the literature search is shown in Fig. 1.

### Methodological quality assessment

The PEDro score evidenced some limitations of the methodological quality assessment: the overall lack of randomization and blinding methods. Points of strength are represented by the good analysis, along with the adequate duration of the follow-up, both performed by most of the included papers. Concluding, the overall quality assessment was good, scoring 6.7 points. The PEDro score related to each study is shown in Tables 1 and 2.

**Table 1:** Demographic baseline of studies included concerning the isolated MPFL reconstruction and related PEDro score

Author, year	Study design	PEDro score	Knees (*n*)	Mean age	Follow-up (months)
Amin et al. 2015 [64]					24.0
Astur et al. 2015 [65]	Prospective	8	30	31.1	60.0
Astur et al. 2015 [65]	Prospective	8	28	28.3	60.0
Ballal et al. 2018 [66]	Prospective	7	20	24.4	12.0
Biondi Pinheiro et al. 2018 [67]	Retrospective	7	16	27.1	31.2
Biondi Pinheiro et al. 2018 [67]	Retrospective	7	21	26.4	34.8
Bitar et al. 2011 [68]	Prospective	8	21	NR	24.0
Bitar et al. 2012 [69]	Prospective	7	56	23.0	19.3
Calapodopulos et al. 2016 [70]	Prospective	5	22	23.1	30.0
Christiansen et al. 2008 [71]	Prospective	6	32	22.0	22.0
Csintalan et al. 2013 [72]	Retrospective	5	56	4.3	51.0
Deie et al. 2011 [73]	Retrospective	5	31	22.2	39.0
Feller et al. 2014 [74]	Retrospective	5	26	24.4	42.0
Fink et al. 2014 [75]	Prospective	7	17	21.5	12.0
Gomes et al. 1992 [76]	Retrospective	5	30	28.0	39.0
Gomes et al. 2004 [77]	Prospective	6	16	26.7	60.0
Gomes et al. 2008 [78]	Prospective	7	12	19.3	53.0
Gomes et al. 2008 [78]	Prospective	7	12	19.0	53.0
Goncaives et al. 2015 [79]	Prospective	6	22	28.6	26.2
Goyal et al. 2013 [80]	Retrospective	5	32	25.0	38.0
Hiemstra et al. 2017 [81]	Retrospective	5	155	25.4	24.4
Hinterwimmer et al. 2013 [82]	Retrospective	6	19	23.0	16.0
Howells et al. 2012 [83]	Prospective	7	155	26.0	16.0
Kang et al. 2013 [84]	Prospective	8	82	28.8	24.0
Kim et al. 2015 [85]	Retrospective	6	9	24.6	19.3
Kita et al. 2015 [86]	Prospective	7	44	25.4	39.0
Krishna Kumar et al. 2014 [87]	Prospective	7	30	18.0	25.0
Lind et al. 2016 [88]	Prospective	8	24	12.5	39.0
Lind et al. 2016 [88]	Prospective	8	179	23.0	41.0
Lippacher et al. 2014 [13]	Retrospective	7	68	18.3	24.7
Malatray et al. 2019 [53]	Prospective	9	16	28.0	24.0
Niu et al. 2017 [89]	Prospective	7	30	25.0	55.1
Panni et al. 2011 [90]	Retrospective	5	48	0.3	33.0
Ronga et al. 2009 [91]	Prospective	5	37	28.0	37.0
Sadigursky et al. 2016 [92]	Prospective	7	31	29.4	12.0
Slenker et al. 2013 [93]	Retrospective	6	35	20.6	21.0
Sillanpaa et al. 2008 [94]	Retrospective	6	18	20.2	121.2
Smith et al. 2014 [95]	Retrospective	6	21	23.0	12.0
Thaunat et al. 2007 [96]	Retrospective	5	23	22.0	28.0
Wagner et al. 2013 [7]	Prospective	6	50	19.0	12.0

**Table 2:** Demographic baseline of studies included concerning the MPFL reconstruction in combination with LRR and related PEDro score

Author, year	Study design	PEDro score	Knees (*n*)	Mean age	Follow-up (months)
Ahmad et al. 2009 [17]	Retrospective	5	20	23.0	31.0
Han et al. 2011 [18]	Retrospective	6	59	24.3	68.4
Kumahashi et al. 2012 [97]	Prospective	6	5	13.6	27.8
Kumahashi et al. 2016 [98]	Prospective	7	17	22.0	45.0
Li et al. 2014 [19]	Prospective	7	65	29.4	78.5
Lin et al. 2015 [20]	Retrospective	5	18	NR	35.0
Ma et al. 2013 [21]	Prospective	8	32	28.4	40.0
Malatray et al. 2019 [53]	Prospective	9	17	28.0	24.0
Matsushita et al. 2014 [56]	Retrospective	6	21	22.1	44.0
Matsushita et al. 2014 [56]	Retrospective	6	18	23.5	38.0
Nomura et al. 2000 [55]	Prospective	7	27	21.0	70.8
Nomura et al. 2006 [22]	Retrospective	6	12	24.8	51.0
Nomura et al. 2007 [23]	Retrospective	5	24	22.5	142.8
Raghuveer et al. 2012 [24]	Prospective	7	15	29.2	42.0
Rhatomy et al. 2019 [99]	Retrospective	7	8	20.0	24.0
Suganuma et al. 2016 [25]	Retrospective	6	18	20.7	51.6
Suganuma et al. 2016 [25]	Retrospective	6	28	20.3	48.0
Vavalle et al. 2016 [100]	Retrospective	5	16	22.0	38.0
Wang et al. 2010 [101]	Retrospective	7	28	29.0	42.0
Wang et al. 2016 [33]	Retrospective	6	26	26.3	38.2
Wantabe et al. 2008 [57]	Retrospective	7	29	19.0	51.6
Witonski et al. 2013 [102]	Prospective	7	10	27.2	43.0
Zhang et al. 2019 [54]	Prospective	7	60	21.0	96.0

### Patient demographic

Data from a total of 2131 procedures were collected. The mean follow-up was 40.87 ± 24.1 months. In the combined group, a total of 585 patients with a mean age of 23.4 ± 4.1 years were included. In the isolated group, a total of 1599 knees with a mean age of 22.1 ± 6.2 years were included. No significant discrepancies were denoted concerning the age at baseline (*P* = 0.2). Table 1 and 2 show the demographic baseline data of the study groups.

### Outcomes of interest

All PROMs of interests improved in favour of the combined group: Kujala (MD  3.8; *P* = 0.01), Lysholm (MD  4.2;*P* = 0.004), Tegner (MD 0.8; *P* = 0.04), IKDC MD 9.8; *P* = 0.02). The ROM resulted comparable among the groups (*P* = 0.4). The main results of the PROMs and ROM at last follow-up are shown in detail in Table 3.

**Table 3:** Mean values of the PROMs and ROM at last follow-up

Endpoint	Isolated group		Combined group		MD	*P*
	Mean ± SD	95% CI	Mean ± SD	95% CI		
Kujala	86.9 ± 6.1	84.94–88.76	90.7 ± 5.3	88.38–93.02	3.8	0.01
Lysholm	88.1 ± 4.9	86.79–89.35	92.3 ± 2.7	91.15–93.49	4.2	0.004
Tegner	4.9 ± 0.7	4.71–5.13	5.7 ± 1.1	5.16–6.16	0.8	0.04
IKDC	75.7 ± 4.7	74.19–77.17	85.5 ± 5.1	83.25–87.69	9.8	0.02
ROM	134.7 ± 10.1	131.28–138.02	133.8 ± 7.9	130.39–137.27	− 0.9	0.4

Between the two groups, similarity was found in terms of positivity to the apprehension test (OR 1.8; 95% CI: 0.9–3.1; *P* = 0.05), rate of complications (OR 1.1; 95% CI: 0.7–1.7; *P* = 0.1), re-dislocations (OR 0.3; 95% CI: 0.1–1.0; *P* = 0.8) and revision surgeries (OR 0.5; 95% CI: 0.3–1.1; *P* = 0.1).

## Discussion

According to the main findings of the present systematic review, MPFL reconstruction combined with LRR reported greater Kujala, Lysholm, and Tegner scores compared to the isolated procedure. However, the improvements of these aforementioned PROMs did not reach the minimal clinically important difference (MCID), and thus their clinical relevance is questionable [38, 40–42]. The rate of overall complications, positivity to the apprehension test, revision surgeries, and re-dislocations were comparable among the two cohort, as well as the range of motion of the knee.

LRR was widely undertaken for a relatively long period. Merchant and Mercer in 1974 published their results on LRR in 16 patients suffering from recurrent subluxations or dislocations, reporting satisfying results in patients with chondromalacia and malalignment [30]. LRR became standard surgery for patellofemoral instability and anterior knee pain, yielding satisfying results [43–47]. At present, however, these indications are outdated, and in the past 15 years, the role of isolated LRR has been revised extensively. At present, the indication for isolated LRR remains imbalance of the extensor mechanism during contraction of the quadriceps muscle group, when excessive tension of the lateral retinaculum exists. Patients suffering from anterior knee pain from lateral hyperpressure syndrome (LHPS) with evidence of patellar tilt are suitable for an isolated LRR [14, 34, 48, 49]. Herein, we remarked the role of the lateral retinaculum lengthening (LRL) [50]. In patients with LHPS, LRL produces less medial instability and quadriceps atrophy with better clinical outcomes at 24 months compared with LRR [51].

In comparison with normal intact knees, an isolated LRR will reduce the forces required to displace the patella 10 mm laterally approximately by 10–20% within the range from 0° to 30° flexion [26, 27]. Furthermore, a LRR will probably re-centralize the patella on its physiological axis. At the same time, however, LRR increases patellar instability by decreasing the pressure in the lateral patellar compartment. Thus, LRR reduces the ability of dynamic stabilizers to maintain patellar tracking and increase role of the anatomical factors that predisposed the patellar to the first dislocation. Therefore, even if patellar tracking may be centralized following LRR, the tendency to lateralization is increased.

The role of combination of MPFL reconstruction and LRR is not entirely clarified. The MPFL is nearly always damaged after the first dislocation, leading to joint instability and a tendency of patella lateralization. Combining LRR with MPFL reconstruction is controversial, and the indications have not been entirely clarified.

Regarding the studies included in the combined group, we must accentuate that few authors performed a LRR in all of their patients [18, 21, 52, 53]. Lateral release has been performed with heterogeneous criteria, and the surgical indications are controversial. Han et al. [18] combined routinely a LRR because they hypothesize that this will improve the post-operative ROM. LRR was performed when lateral retinaculum over-tension (defined as force directed medially being less than one fourth of the force directed laterally) was noticed [32]. Other authors performed an LRR in patients with moderate patellar maltracking or excessive tightness or stiffness of the soft tissue structures lateral to the patella [22–25, 32, 33, 54–57]; however, quantification of these variables has not been undertaken. Other authors [17, 19, 20] evaluated the stability of the lateral structures by everting the patella: if unable to evert the patella, a LRR was performed. Regardless of the criteria used to indicate a LRR, its role in the management of patellar instability is still unclear and debated. We hypothesize that the reduced lateral compression within the articular surface explain the better results observed in the LRR group. In fact, the lateral patellar facet slides over the lateral trochlea and subsequently over the lateral femoral condyle: in patellar dislocation, the trauma causes chondral and osteochondral defects in 50–96% of cases, especially after the first dislocation [58–63].

The present study has several limitations. First, the studies performing isolated MPFL reconstruction were far more numerous than those performing a combined intervention. Another important limitation is the overall low quality of studies included. To improve data pooling, both prospective and retrospective studies were included in the analysis, representing another potential limitation of the present study. Further studies should improve the evidence on this topic, improving the quality of their methodology. Further studies should also clarify the role of lateral retinaculum lengthening. The analyses were performed regardless to the type of graft (e.g. semitendinosus, gracilis, quadriceps), the number of bundle (single or double), and the presence of additional risk factors (e.g. patella alta, elevated TT-TG distance, genu valgum) that may influence the surgical outcomes. Given these limitations, data must be interpreted with caution. Strengths of the present work were the comprehensive nature of the literature search, along with the strict eligibility criteria and the adequate baseline comparability. To the best of our knowledge, this investigation involves the highest number of studies and procedures, thus representing another important point of strength. Given the lack of evidence and heterogeneous indications, future studies should investigate which cohort of patients can benefit from a LRR.

## Conclusion

According to the main results of this systematic review, there is no evidence of a clinically relevant difference between the two procedures. On this basis, isolated MPFL reconstruction should be recommended. There is no evidence that adding a lateral release impacts positively on the outcome of MPFL reconstruction. The role of lateral retinacular lengthening should be studied more extensively.
